# Assessment of holographic microscopy for quantifying marine particle size and concentration

**DOI:** 10.1002/lom3.10379

**Published:** 2020-08-05

**Authors:** Noah L. Walcutt, Benjamin Knörlein, Ivona Cetinić, Zrinka Ljubesic, Suncica Bosak, Tom Sgouros, Amanda L. Montalbano, Aimee Neeley, Susanne Menden‐Deuer, Melissa M. Omand

**Affiliations:** ^1^ University of Rhode Island, Graduate School of Oceanography Narragansett Rhode Island USA; ^2^ Brown University, Center for Computation and Visualization Providence Rhode Island USA; ^3^ NASA Goddard Space Flight Center Ocean Ecology Laboratory Greenbelt Maryland USA; ^4^ GESTAR/Universities Space Research Association Columbia Maryland USA; ^5^ Faculty of Science, Department of Biology University of Zagreb Zagreb Croatia; ^6^ Department of Computer Science Brown University Providence Rhode Island USA

## Abstract

Holographic microscopy has emerged as a tool for in situ imaging of microscopic organisms and other particles in the marine environment: appealing because of the relatively larger sampling volume and simpler optical configuration compared to other imaging systems. However, its quantitative capabilities have so far remained uncertain, in part because hologram reconstruction and image recognition have required manual operation. Here, we assess the quantitative skill of our automated hologram processing pipeline (CCV Pipeline), to evaluate the size and concentration measurements of environmental and cultured assemblages of marine plankton particles, and microspheres. Over 1 million particles, ranging from 10 to 200 *μ*m in equivalent spherical diameter, imaged by the 4‐Deep HoloSea digital inline holographic microscope (DIHM) are analyzed. These measurements were collected in parallel with a FlowCam (FC), Imaging FlowCytobot (IFCB), and manual microscope identification. Once corrections for particle location and nonuniform illumination were developed and applied, the DIHM showed an underestimate in ESD of about 3% to 10%, but successfully reproduced the size spectral slope from environmental samples, and the size distribution of cultures (*Dunaliella tertiolecta*, *Heterosigma akashiwo*, and *Prorocentrum micans*) and microspheres. DIHM concentrations (order 1 to 1000 particles ml^−1^) showed a linear agreement (*r* ^2^ = 0.73) with the other instruments, but individual comparisons at times had large uncertainty. Overall, we found the DIHM and the CCV Pipeline required extensive manual correction, but once corrected, provided concentration and size estimates comparable to the other imaging systems assessed in this study. Holographic cameras are mechanically simple, autonomous, can operate at very high pressures, and provide a larger sampling volume than comparable lens‐based tools. Thus, we anticipate that these characterization efforts will be rewarded with novel discovery in new oceanic environments.

Quantitative marine particle measurements, such as size‐spectral shape and particle concentration, provide insights into ecological community composition, abundance, diversity, and biogeochemical processes (Lombard et al. 2019). However, the discrete quantification and classification of individual plankton, marine snow, and other detritus (herein referred to as marine particles) over the depth and lateral expanse of the ocean persists as a methodological challenge (Boss et al. 2015), in part because there is not a perfect tool that can achieve broad coverage while also capturing a wide range of marine particle size classes (Stemmann and Boss 2012).

A promising technology for addressing this challenge is holographic microscopy (Jericho et al. 2013; Talapatra et al. 2013; Yourassowsky and Dubois 2014; Zetsche et al. 2014; Göröcs et al. 2018). Holographic microscopy can record entire volumes of water simultaneously, and because of this ability, comparatively large volumes can be sampled per frame (Watson 2018). The holographic microscope used in this study, a 4‐Deep HoloSea (herein referred to as DIHM), samples 100X larger volumes than comparable objective lens‐based systems (Menden‐Deuer et al. 2020), 0.1 mL per image. They capture a wide range of size classes (10–2000 *μ*m in diameter) because the image is not constrained by a depth‐of‐field, given that image focusing occurs in postprocessing (Xu et al. 2001; Schnars and Jüptner 2005). In contrast, traditional microscopes sacrifice greater depth‐of‐field for higher magnification. In addition, these instruments are capable of operating in situ in a variety of deployment configurations (such as CTD‐rosette, flow‐through, and autonomous systems) without intake pumps, which means less disturbance to the delicate morphology of large, rare particles. This handling in turn affects the observed size of particles (Bochdansky et al. 2016), the natural association of particles, and also the motions of particles within the sample volume. An advantage of recording relatively undisturbed motions of particles in 3D is that fluid diagnostics can be developed (Katz and Sheng 2009) for shedding new insight on physical–biological interactions (Katz et al. 1999; Nayak et al. 2018). Lenses and moving parts are prone to mechanical failure, and because these instruments operate without them, a lowered rate of mechanical failure may eventually translate to longer deployment lengths, more ocean sampled, and in turn greater statistical confidence in ecological and biogeochemical assessments.

To evaluate the utility of digital holographic microscopy for measurements of aquatic particle size abundance spectra, we offer a review of the literature that is relevant to the topic of this paper. However, it is important to note that only few lab‐ and field‐based experiments, focusing on concentration and PSD, were found relevant to our study, due to the specificities of the method, elaborated in previous paragraphs. Graham and Nimmo‐Smith (2010) found good agreement between the Holographic Particle Imager and the Malvern Hydro 2000G laser sizer for grains of sand 125 to 700 *μ*m in diameter. When they later expanded this assessment to include in situ biological data (6.75–500 *μ*m in equivalent diameter) for the holocam compared alongside the LISST‐100X laser diffraction particle sizer, results of the size and PSD intercomparisons were mixed (Graham et al. 2012). Guildenbecher et al. performed a series of lab‐based intercomparison experiments using a custom digital inline holographic microscope (DIHM) setup for size and concentration of silicon microspheres (100–1000 *μ*m in diameter) in oil, and compared these to measurements made in parallel from a Malvern Mastersizer and found agreement to within 4% of the actual values (Guildenbecher et al. 2013). While these size results are informative for understanding the theoretical limits of the size and concentration accuracy under ideal conditions, marine scientists should be cautious to extrapolate the accuracy from a simple spherical geometry to more complex and highly varied geometry of oceanic particles (Gabas et al. 1994; Kelly et al. 2006). Where the aforementioned results are relevant to collimated‐light source holographic microscopes, Bochdansky et al. performed a series of laboratory‐based and field‐based holographic microscope assessments for a point‐source configuration (Bochdansky et al. 2013). These results validate sizing accuracy of point source DIHM and provide qualitative assessment of DIHM surface concentration measurements of *Trichodesmium* colonies.

This study compares the DIHM with multiple different particle sizing instruments, evaluating both lab‐ and field‐based measurements, and analyzing a diversity of particle morphologies and concentrations. Amid the growing variety of image‐based particle sizing techniques, intercomparability exercises are critical for improving the standards of marine ecological assessments via analogous observations (Reynolds et al. 2010; Lombard et al. 2019).

## *Methods*

**Fig 1:**
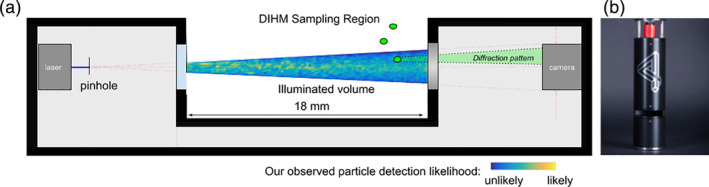
(**a**) Schematic drawing of the DIHM illustrates how the watertight housing encloses the optical setup. At a high level, the optical setup is comprised of a laser source, pinhole, two glass windows, and a camera. Note that no lenses are needed for focusing. Laser light travels from the laser, through the pinhole, into the imaging volume, and finally onto the camera face. The geometry of the DIHM illumination cone is shown to scale (red dashed lines). The illumination volume is unobstructed such that particles (shown as green circles) can freely flow into this illumination and be detected via their constituent diffraction patterns. Particle detection likelihood by the Brown University Center for Computation and Visualization's Hologram Processing Pipeline (CCV Pipeline) are overlaid onto the imaging volume, illustrating how particle detection likeliness is nonuniform. The highest detection probabilities are closest to the laser (yellow regions) and the lowest detection probabilities are closest to the camera and far from the central axis (blue regions). A simplified and exaggerated cartoon of the particle‐light interaction is also shown (green shaded region), where particle diffraction patterns magnify in size across space before reaching the CCD camera sensor (green shaded region). (**b**) The 4‐Deep HoloSea (image printed with permission from 4‐Deep Inwater Imaging).

The digital inline holographic microscope (DIHM) used for this study, a 4‐Deep HoloSea,^1^ is an inline, point‐source holographic microscope with a source to camera distance of 10 cm, a pinhole size of 0.5 *μ*m, and a view‐able focal depth of 1.8 cm. We computed the working imaging volume (i.e., region illuminated by the laser) as 0.063 mL. The illumination and image acquisition consist of a 386 nm laser source and a 1 inch charge‐coupled device (CCD) camera (IMPERX BOBCAT B2020), which can capture the raw holograms (2056 × 2060 pixels, 7.4 *μ*m per pixel, 4 MB per hologram) at a maximum of 22 frames per second (fps) for up to 250 GB of solid state memory, or roughly 60,000 images for uncabled deployments. This illumination casts diffraction patterns around particles that intercept the beam (Fig. 1). A camera records the light intensities of the diffraction pattern in 2D, and this pattern is later solved for wave front intensity for all points in the conically shaped imaging volume. Solutions of point intensities require knowledge of the laser's wavelength and the distance from laser to camera, or path‐length (Garcia‐Sucerquia et al. 2006). Parsing the planes along the laser's path‐length in the imaging volume is similar to turning the focusing knob of a light microscope, and yields an in‐focus “reconstructed” plane at the z‐location where the object was located. A watertight camera housing and external data logger with battery pack enable deployments to a maximum depth of 2000 m, and fiber optic connections make it possible to rapidly recover the raw hologram data (e.g., in between CTD rosette‐based deployments).

After deployment, raw hologram acquisition and reconstructions were performed by the 4‐Deep's Octopus software, which numerically solves point source wave front intensities for an object's 2D diffraction pattern using the Kirchhoff–Helmholtz transform (Kreuzer et al. 1992; Garcia‐Sucerquia et al. 2006). This GPU‐enabled algorithm rapidly reconstructs hologram image planes.

However, a major challenge in acquiring in‐focus contours for ecological analysis is that the optimal focal depths for any particular particle are not known prior to the reconstruction steps (Katz and Sheng 2009). Reconstruction steps have previously required time‐consuming, manual identification as tens of thousands of holograms are recorded per hour of deployment. In an effort to accelerate the process of detecting, segmenting, and extracting focused marine particle contours from holograms, various workflows for unsupervised, that is, automated analysis of holograms have been developed. Malkiel et al. developed some of the first workflows for large (480 cm^3^, 35 GB/hologram), emulsion‐based holographic volumes (Malkiel et al. 2004). Burns and Watson (2007) developed the HoloCruncher workflow for digital holograms, specifically those recorded by the eHoloCam. More recently, HoloProc^2^ was developed for users of the commercially available LISST‐Holo system,^3^ and MATLAB libraries are available to download (Davies et al. 2015). The 4‐Deep Stingray software^4^ automates the analysis steps and can be used either during real‐time data collection or in an offline mode for previously recorded data. Above‐mentioned packages are mostly relevant to collimated‐source holographic microscopes, and the source code is closed such that modifications cannot be made to adapt and fine tune the workflow for the analysis of holograms from point‐source holographic microscopes.

**Fig 2:**
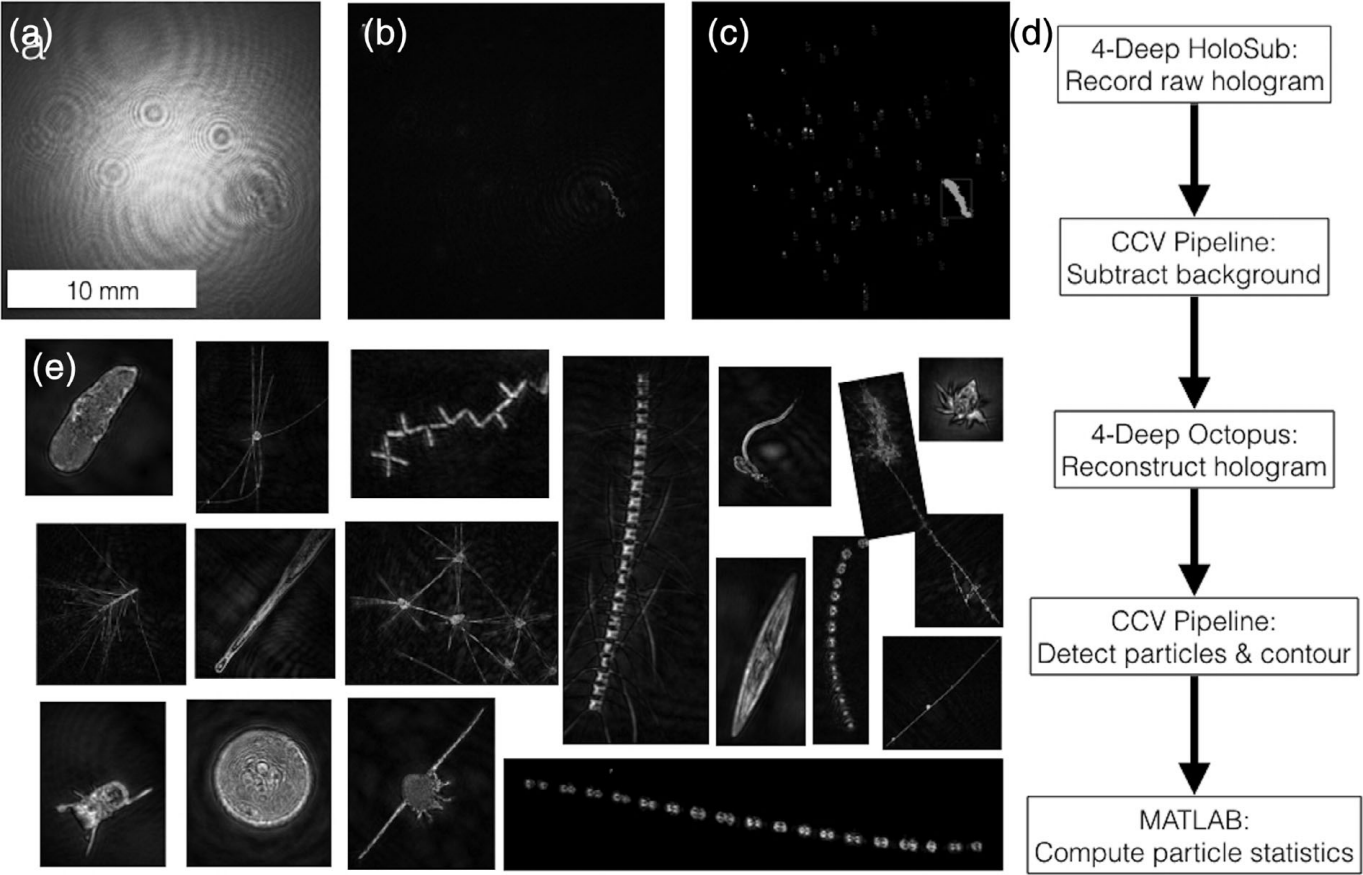
The custom hologram processing pipeline extracts 2D contours from the image volume. (**a**) A raw 2D hologram. (**b**) A refocused hologram image at 16,250 *μ*m from the laser source, revealing chain‐forming diatoms. (**c**) Regions of interest (colored and sorted) derived from the image processing pipeline. (**d**) A flowchart of the hologram processing pipeline illustrates the role of each constituent software package, from raw hologram to quantitative particle statistics. The 4‐Deep HoloSea and Octopus software record the raw holograms. The CCV Pipeline computes and subtracts the image background, which is calculated as a composite of median pixel intensities for three images before and after the image of interest. This step is critical for field deployments in which small contaminants frequently adhere to optical surfaces and lead to repeated construction of particles stuck to the instrument. The 4‐Deep Octopus software reconstructs background‐subtracted holograms. The CCV Pipeline detects and contours particles. MATLAB code refines data quality and computes particle statistics. (**e**) An assortment of re‐focused hologram contours illustrate a variety of large marine “particles” (phytoplankton, zooplankton, pellets and detritus) with sizes ranging from 10 to 200 *μ*m ESD.

The CCV Holographic Image Processing Pipeline (Fig. 2, herein referred to as the CCV Pipeline^5^), developed by our group, is an unsupervised analysis package relevant to point‐source holographic microscopes that automates the process of determining the optimal focal depths of particles from the 4‐Deep Octopus software for high‐throughput hologram analysis (Walcutt et al. 2019). First, the CCV Pipeline saves slices of the hologram (i.e., focal planes) from Octopus in user‐specified *μ*m increments across the 22 mm path between the point source and the camera. Next, a sharpness score is computed for each pixel in all image planes and the maximum value is stored, a technique alike ones previously developed (Guildenbecher et al. 2012; İlhan et al. 2014). As neighboring in‐focus pixels are likely to belong to the same object, pixels are grouped to segments in the second step of the processing (Suzuki and Be 1985). For each segment, the optimal focus distance is computed based on the same sharpness score used in the first step, but for the whole region (*see* Fig. 2c). Finally, the image is refocused for each segment at the optimal distance, and the particle is segmented using the GrabCut algorithm (Rother et al. 2004). This results in a 2D representation (*see* Fig. 2d) for each particle as well as its three‐dimensional position within the volume.

After in‐focus particle contours were extracted from the hologram using the CCV Pipeline, postprocessing steps were developed to remove image artifacts. This algorithm was based on region of interest (ROI) z‐depth sharpness score, and was manually calibrated and tested for our setup. This procedure was similar to previously developed methods (Mallahi and Dubois 2013; Gao et al. 2014). While we experimented with other artifact detection methods, such as artifact detection using object pixel intensity variance, we found this method yielded results that were less sensitive to empirically determined thresholds for a range of object sizes. Finally, steps were taken to improve quality of the contour drawn around the ROIs by applying an edge sharpening convolution, such that a single, continuous, and solid shape could be extracted for more accurate ROI statistics.

With the possibility of detecting, segmenting, and extracting marine particles from large volumes of hologram data, we designed a series of experiments to evaluate the quantitative skill of our software package. Marine particle size (quantified through equivalent spherical diameter [ESD]) and concentration are baseline ecological diagnostics (Cavender‐Bares et al. 2001), and provide a straightforward means of quantitative evaluation. While calibrated microspheres are frequently used as a standard for the evaluation of size estimation performance (for image‐based techniques), no such gold standard exists for evaluating concentration estimates. In the absence of this standard, we designed a series of experiments to evaluate concentration estimates by comparing the relative agreement from analogous measurement techniques. Here, we assume that agreement between two or more different techniques suggests convergence on the true concentration, and weak agreement between multiple different techniques suggests low accuracy or systematic bias in the measurement scheme or setup.

Our experimental approach was inspired by the use of continuous and analogous marine particle sampling tools used during cruise FK170124 aboard the R/V Falkor (Sea to Space Particle Investigation 2017‐01‐24 to 2017‐02‐20). These image‐based, particle sizing techniques included: the McLane Research Laboratories Imaging FlowCytobot (IFCB; Olson and Sosik 2007), the Fluid Imaging Technologies FlowCam (FC; Sieracki et al. 1998), and manual microscope counts (Utermöhl 1931).

With a means to quantitatively assess size and concentration estimates via intercomparison, we prepared five different suspended particle types for laboratory‐based processing. Particle types and densities used for this study were selected to simulate those found in the surface ocean for a wide range of environments. Oligotrophic gyres have been observed to have particle concentrations of 10 particles mL^−1^ for cell sizes, ranging from 1 to 10^2^ *μ*m ESD (White et al. 2015). On the opposite end of the spectrum, nearshore bloom conditions have been observed with particle concentrations as high as 10^7^ particles mL^−1^, for cell sizes and cell chain lengths ranging from 1 *μ*m to several millimeters (Karentz and Smayda 1998). Bacteria are not reliably identifiable using this system and therefore excluded from this analysis. To avoid the pitfalls of shape‐specific measurement bias, a variety of cell forms and sizes were selected including spherical, elongate, and asymmetrical. Use of cosmopolitan representatives from major phytoplankton groups further reduced the possibility of any organism‐specific size or concentration biases. The monocultures used in this experiment included a green algae *Dunaliella tertiolecta*, raphidophyte *Heterosigma akashiwo*, and dinoflagellate *Prorocentrum micans*.

*Heterosigma akashiwo* and *Prorocentrum micans* were grown and processed ashore in autoclaved, 0.2 *μ*m sterile‐filtered amended seawater with f/2 medium without silica (Guillard 1975). These cultures were not axenic. Cells were maintained at a light level of 80 to 100 *μ*mol photons m^−2^ s^−1^ and a 12 : 12 h light : dark cycle at 15°C. *Dunaliella tertiolecta* were grown and processed aboard the R/V Falkor in ambient light and at room temperature. Field samples (which include whole plankton communities) were collected via Niskin bottles at FK170124 Sta. 2, CTD deployment 12 (27.71°N, 139.50°W) in the North Pacific.

While field samples were processed directly from Niskin Bottles aboard the R/V Falkor using different depths to capture a range in particle densities and morphologies, laboratory monoculture and microspheres^6^ were diluted and analyzed in seawater filtered using a 0.2 *μ*m glass fiber filter. After preparation and immediately prior to processing, samples were resuspended using 30 s of inversions. Sample preservation occurred rapidly after preparation to avoid excess growth prior to processing. When not immediately available for processing, live monocultures were preserved in either Lugol's solution (*Heterosigma akashiwo* and *Prorocentrum micans*) or with 36% formaldehyde solution (*Dunaliella tertiolecta* and environmental samples). Multiple runs and counts on each sample provided statistical confidence and means for uncertainty quantification.

### DIHM methods

To analyze these samples in the DIHM, the sampling region (Fig. 1) was sealed from the outside using Parafilm wrap,^7^ and a 75 mL sample was injected into the sampling region. The injected samples were agitated gently with the pipette to de‐bubble the solution and also avoid particle clumping and settling. Prior to recording, the detector's exposure time was adjusted within the Octopus software to create a uniform distribution of light intensities. Next, the DIHM recorded at 16 fps for a minimum of 6 min. After recording, the walls of the sampling region were thoroughly scrubbed and rinsed with de‐ionized water to prevent cross‐contamination between samples. In addition, the external lenses of the DIHM were wiped clean using lens paper and isopropyl alcohol. The CCV Pipeline foreground‐background threshold parameter was adjusted to 0.95 for all samples to achieve sharp, optimally segmented particles. Although fixed intensity thresholds for object detection were applied here, these results could be further improved with local‐adaptive thresholds. This enhancement to object detection, in which the threshold for object detection varies as a function of background–foreground illumination intensity level, would help compensate for nonuniform backgrounds. Size was assessed as ESD from these segmented particles using the MATLAB Image Processing Toolbox,^8^ which translates the surface area (SA) of irregularly shaped 2D image contours to standardized circular objects of equivalent SA using the formula $\mathrm{ESD}=2\times \sqrt{\left(\mathrm{SA}/\pi \right)}$. Particle concentration was assessed as overall concentration, which we computed as the total particles counted for a given sample divided by the total volume sampled. The total volume sampled by the DIHM was computed as the number of analyzed holograms multiplied by the volume of an individual hologram. The latter two variables, total particles counted and individual hologram volume, although seemingly straightforward, proved challenging to accurately interpret from the DIHM and CCV Pipeline and will be discussed at greater length in the Results section (below).

### FlowCam methods

All samples were imaged with a 10X objective lens and set to run in the instrument's trigger mode setting. Manual focusing of the objective on test run particles provided crisp contours for measurement. The flow cell was selected to image particles that ranged from 1 to 100 *μ*m, with a minimum distance between particles of 1 *μ*m. An 80 *μ*m flow cell was used for smaller monocultures (*Dunaliella tertiolecta*, *Heterosigma akashiwo*, and *Prorocentrum micans*), while a 300 *μ*m flow cell was used to capture larger particles from the environmental samples. The flow rate was adjusted between 0.1 and 0.15 mL min^−1^ to optimize particle detection from the light scattering and fluorescence triggers in the instrument's trigger mode, which minimized duplicate images from being recorded. The frame rate varied between 0.04 and 1 fps. Care was taken to purge the flow cell and flow cell tubing before beginning the next sample run, by pumping filtered seawater through the system a minimum of three times, and manually inspecting the camera view for the presence of any residual particles. These settings varied between culture type, but large modifications were avoided between sample runs for a given culture. Image segmentation and feature extraction were computed using the VisualSpreadsheet® Particle Analysis Software^9^ versions 3.2.3 (*Dunaliella tertiolecta* and environmental samples) and 4.0.27 (all other samples). Particle sizes were computed as ESD. Particle concentration was computed by dividing the fluid volume imaged by the total particles imaged.

### Imaging FlowCytobot methods

All samples were imaged with a 10X objective lens. After priming, optical focus of objects was tested and manually focused to provide optimal clarity. Laser‐induced fluorescence is used to generate a trigger for image acquisition. The flow rate of the IFCB was fixed at 0.25 mL min^−1^. The flow cell of the IFCB enables imaging of particles between 3 and 300 *μ*m. Care was taken to purge the flow cell and flow cell plumbing before beginning each sample run by priming the IFCB in the de‐bubbling mode a minimum of three times, and inspecting the camera view for the presence of any “leftover” particles. These parameters varied between culture type, but large modifications were avoided between sample runs for a given culture. Image segmentation and feature extraction was performed using the ifcb‐analysis MATLAB package.^10^ This package utilizes the MATLAB Image Processing Toolbox to compute particle size as ESD.

### Manual microscope counting methods

For this study, only the observed overall concentration is intercompared with the other concentration estimation methods (Lund et al. 1958; Karlson et al. 2010). An inverted Zeiss Axiovert 200 (Carl Zeiss, Oberkochen, Germany) microscope equipped with a bright‐field optics, phase contrast, and Nomarski differential interference contrast was used to count the cells. The variable volume (10 mL for cultures or 50 mL for environmental samples) was sedimented in Utermöhl combined plate counting chambers (HydroBios, Kiel, Germany) and analyzed after > 24 h according to Utermöhl technique (Utermöhl 1931; Lund et al. 1958). Cells from cultures and microspheres were counted on a variable number (5–10) of random chosen fields at 400X magnification, or on half of the counting chamber bottom. Cells in environmental samples were counted at two transects along the counting chamber bottom at 400X and 200X magnification, depending on their concentration, as well as on the bottom half of the chamber at a lower magnification of 100X, to obtain a more correct evaluation of less abundant plankton taxa.

### Size intercomparison methods

For the three automated particle sizing methods (DIHM, IFCB, and FC), size was intercompared as size‐specific concentration. Size‐specific concentration was computed as the magnitude of each particle size class normalized by the width of that size class bin. The minimum bin‐width used for analysis was 3 *μ*m. This parameter was determined by the maximum optical resolution reported by the respective instrument manufacturers: 1.5 *μ*m for the DIHM,^11^ 3.4 pixels per micron for the IFCB,^12^ and 3 *μ*m for the FC.^13^ Bin‐widths were evenly spaced at a constant 3 *μ*m for the microspheres and monoculture, and logarithmically spaced bin‐widths were used for the environmental samples. The minimum bin size center was 3.5 *μ*m ESD (for the *Dunaliella tertiolecta*) and the maximum was 120 *μ*m ESD (for the environmental samples). Given that fixatives can alter the size of cells (Booth 1987; Menden‐Deuer et al. 2001) and low accuracy in the micron‐size length scales, sizing results from manual microscope counts were not included in the analysis.

### Statistical analysis methods

For assessing size and concentration, linear regression analysis was used to quantify the relationship between DIHM, FC, IFCB, and microscope counts for all particle types. This approach was applied for all instrument‐instrument comparisons, and for instrument‐overall‐instrument‐concentration mean comparisons. Given that there was error associated with all concentrations measured, Model II regression was applied for particle concentration assessments (Laws and Archie 1981). For particle size assessments, Model I regression was used to obtain the slope of all Niskin‐bottle particle size spectra, since the particle sizes were measured with higher precision than the size‐specific concentrations. For overall comparisons, the mean difference between each instrument/method for size and concentration was computed as the mean of (Instrument A − Instrument B) / Instrument A.

### Size correction method

The particle contours recorded by point‐source, holographic microscopes are magnified projections of the original contour, as the illumination source casts a radially expanding light field across the imaging volume. The recorded contour sizes can be accurately adjusted for this effect by calibrating against microspheres of a known size. Using 50 *μ*m microspheres, we developed the size correction coefficients for the DIHM, and found the measured contour size (*S*_*m*_) of the microspheres are adjusted to the actual size (*S*_corr_, mm) as an exponential function of the distance from the laser source (*D*_*m*_, mm):

(1) $$S_{\text{corr}}=0.121\times S_{m}\times D_{m}^{0.76}$$

This is in contrast to Bochdansky et al. who reported the size‐distance correction function as a linear relationship (Bochdansky et al. 2013). See Supplemental information for calibration curve. The fit of this curve had an R‐squared value of 0.86. This difference (linear vs exponential function) might be attributed to the fact that the Octopus reconstruction algorithm assumes the wave produced by the point source to be perfectly spherical, and any deviation from this may introduce error into the distance‐to‐size correction (Sergey Missan of 4‐Deep, personal communication March 11, 2019).

The range of microsphere values observed by the DIHM, that is, FC, and IFCB was a minimum of 45 and 55 *μ*m (Fig. 4a). The overall mean was 50 *μ*m for each instrument. Size specific concentration varied between 1 and 30 spheres (mL *μ*m)^−1^. To illustrate greater detail in this variation per sample, the y‐axis is not fixed for Fig. 4a1–a5. The DIHM size specific concentration showed the greatest similarity to the IFCB. The FC tended to record lower microsphere size‐specific concentration than the IFCB and DIHM. Overall, the mean observed size for the microspheres by the DIHM, FC, and IFCB were highly accurate, with the deviation of all samples less than 2% of the actual size.

### Concentration correction method

Concentration was computed as the number of observed particles divided by the total volume sampled. The approach to quantifying the total volume sampled varied for the flow cytometric and holographic approaches. The total volume sampled for the IFCB and FC is equal to the volume of the respective 5 and 2 mL syringes used to pump media through the imaging flow cells. The actuation distance for the sampling syringes' drive mechanism outputs actuation length, which can be converted to volume sampled in and be used as verification for the precise volume sampled. The total volume sampled by the DIHM was computed as the number of analyzed holograms multiplied by the volume of an individual hologram. Individual hologram volume, although seemingly straightforward, proved challenging to accurately interpret from the DIHM and CCV Pipeline. In this section, we will discuss the challenges in measuring particle concentration for the DIHM and our strategy for mitigating these challenges by correcting the observed particle count to the “true” particle count. After applying our concentration‐correction strategy, which works independent of external measurement sources or calibrations, we observed good agreement with the IFCB, FC, and manual microscope counts.

The volume of each individual hologram was determined geometrically using the formula *V* = 1/3 × *π* × (*r*_1_^2^ − *r*_2_^2^), where *r*_1_ is the maximum radius of the conical beam that particles could pass through nearest the detector, and *r*_2_ is the minimum radius of the conical beam that particles could pass through nearest the laser source. The values *r*_1_ and *r*_2_ were determined empirically by examining the spatial distributions of the beam “edge” that still illuminated a significant number of particles. While a larger imaging volume (and larger *r*_1_ and *r*_2_ values) can capture more particles and larger particles, this comes at the cost of sampling particles with lower illumination. It has been our experience that particles with lower illumination will have lower contrast and therefore tend to get detected by the CCV Pipeline with lower frequency. Hence, the detection of particles at the edge of the beam (where illumination is relatively low) was markedly low. To compensate for this “fuzzy” boundary, we opted to combine all the observed particle XYZ positions for all sample types and “draw” the edge of the beam. We did so conservatively so as to capture the majority of particles. However, this step introduces an issue inherent to open‐volume holographic sampling systems: that light intensity varies and does not provide an equivalent to a physical stop, such as that provided by a flow cytometer's syringe and flow cell. A beam of light does not constitute a closed cell, and so therefore nonuniform illumination will be a consideration in drawing sampling volume boundaries. As previously discussed, the nonuniform object intensity problem is only partially resolved by user‐defined thresholds and as we will see below, nonuniform illumination impacts the detection of all particles.

The total number of particles counted for a sample can be used to compute the particle concentration if, for a well‐mixed homogeneously distributed solution, we make the assumption that the probability of detecting a particle is equal throughout the volume. If the probability of detection is nonuniform, we will consistently measure inaccurate concentrations as detection‐deficient regions will bias the counts. It has been our experience with raw, uncorrected particle count data from the DIHM (processed by the CCV pipeline), that the probability of detection is nonuniform. This observation is based on the spatial distribution of over 10 million particle XYZ position observations of well‐mixed media (such as monoculture and microspheres, *see* Supplemental Information). Consequently, the concentrations reported by the DIHM and CCV Pipeline are as much as 7X lower than those observed by the IFCB, FC, and microscope counts. Although external concentration calibrations might be developed to adjust the measurements by the DIHM and CCV Pipeline, it is not advisable. Gold standards for concentration calibration are technologically in‐feasible, and furthermore this strategy would be highly sensitive to ongoing instrument‐ and environment‐specific variations.

**Fig 3:**
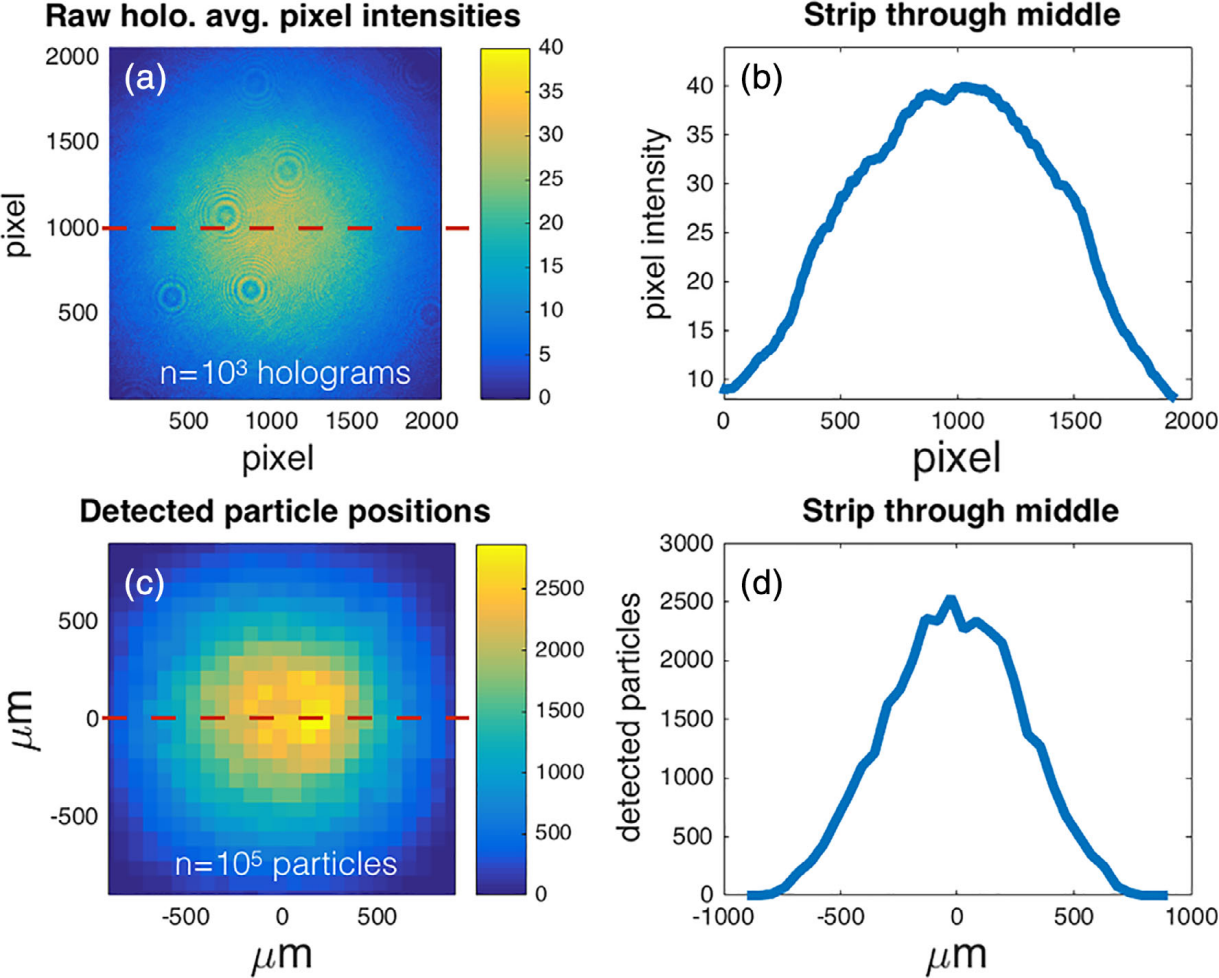
The probability distribution of detected particles in the imaging volume varies horizontally, vertically, and with depth. The frequency of detected particles collapsed onto a single depth plane shows that the greatest number of detected particles occurs at the center of the imaging volume (**c**). A similar distribution in the raw hologram pixel intensity (**a**) suggests that the detection of objects is dependent on the nonuniform intensity of the illumination source. A single slice through the detected particle distribution (red dashed line) shows Gaussian distribution (**d**). Qualitative similarity between a slice in the mean hologram intensities (red dashed line) suggests that the observation probability of uniformly distributed objects is a function of laser intensity.

We have developed a strategy (not previously reported in the literature) to account for the nonuniform probability of particle detection in the DIHM which can improve particle count comparability. This strategy is applicable to any DIHM geometry, and is internally calibrated for a given system (i.e., does not require another instrument to work). It accounts for nonuniform detection probability by modeling detection probability as a function of spatial position relative to the laser source. This model is empirically determined, and for our system was performed by fitting a 2D Gaussian distribution to detection frequency in the XY plane, and then a 1D Gaussian distribution to detection frequency in the ZX and ZY planes for over 10 million particle positions (Fig. 3). The output of this model is a set of scaling coefficients which can be applied to any particle. The scaling coefficients are computed as a function of a given particle's XYZ position, and given by the relationship

(2) $$C\left(x,y,z\right)=e^{-0.5\left({\left(\frac{r}{g_{r}}\right)}^{2}+{\left(\frac{z-z_{o}}{g_{z}}\right)}^{2}\right)}$$

where the scaling coefficient *C* is a function of the distance from the laser source plane to the particle (*z*) and the radial distance (*r*) from the source‐to‐camera axis. The distance *z* is defined as the parallel offset plane from the laser source, and *r* is the radial length from the source‐camera axis along this plane where $r=\sqrt{\left(x^{2}+y^{2}\right)}$. The coefficients *z*_*o*_ (8000 *μ*m), *g*_*r*_ (466 *μ*m), and *g*_*z*_ (6400 *μ*m) were determined by a best fit of these constituent distributions (*see* Supplemental Information).

To apply a region‐specific concentration scaling coefficient to the artifact‐removed data, an observed particle was multiplied by its corresponding integer scaling coefficient (decimal‐value scaling coefficients were rounded down to the nearest integer value). The advantage of effectively replicating a particle of a given size class is that the “shape” of the particle size distribution (PSD) is preserved. For example, a 15 *μ*m particle is observed at the edge of the XYZ imaging space where the scaling coefficient is 3X lower than the most probable region. Hence, three of these particles are effectively added to the list of observed particles (according to the infrequency of observing particles in this region). A given particle could be replicated a minimum of zero times (objects in the center of the beam), and a maximum of seven times (periphery of the beam) with the overall average multiplication factor of 3X. Artifact removal steps were applied prior to the concentration scaling steps. See Supplemental Information for details.

## *Results*

**Fig 4:**
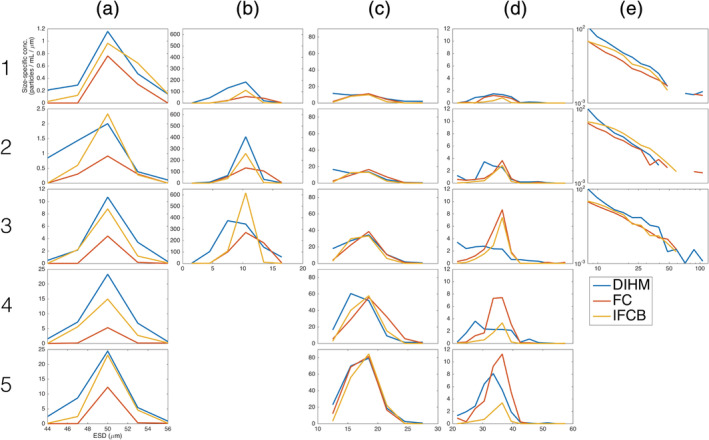
The particle size distribution intercomparison results for the DIHM, FC, and IFCB show good agreement after artifact‐filtering. Results are shown for 50 *μ*m microspheres (**a**), *Dunaliella tertiolecta* (**b**), *Heterosigma akashiwo* (**c**), *Prorocentrum micans* (**d**), and environmental samples (**e**) for the DIHM (blue line), Imaging FlowCytobot (red line), and FlowCam (yellow line). Sample numbers increase in concentration 1 (lowest) to 5 (highest) for microsphere and monoculture samples, and Niskin bottle, that is, environmental sample collection depth. Note that the y‐axis limit is fixed for the *Dunaliella tertiolecta*, *Heterosigma akashiwo*, *Prorocentrum micans*, and environmental sample concentrations, but the y‐axis limit is not fixed for the beads in order to highlight greater detail for the calibration PSD.

**Fig 5:**
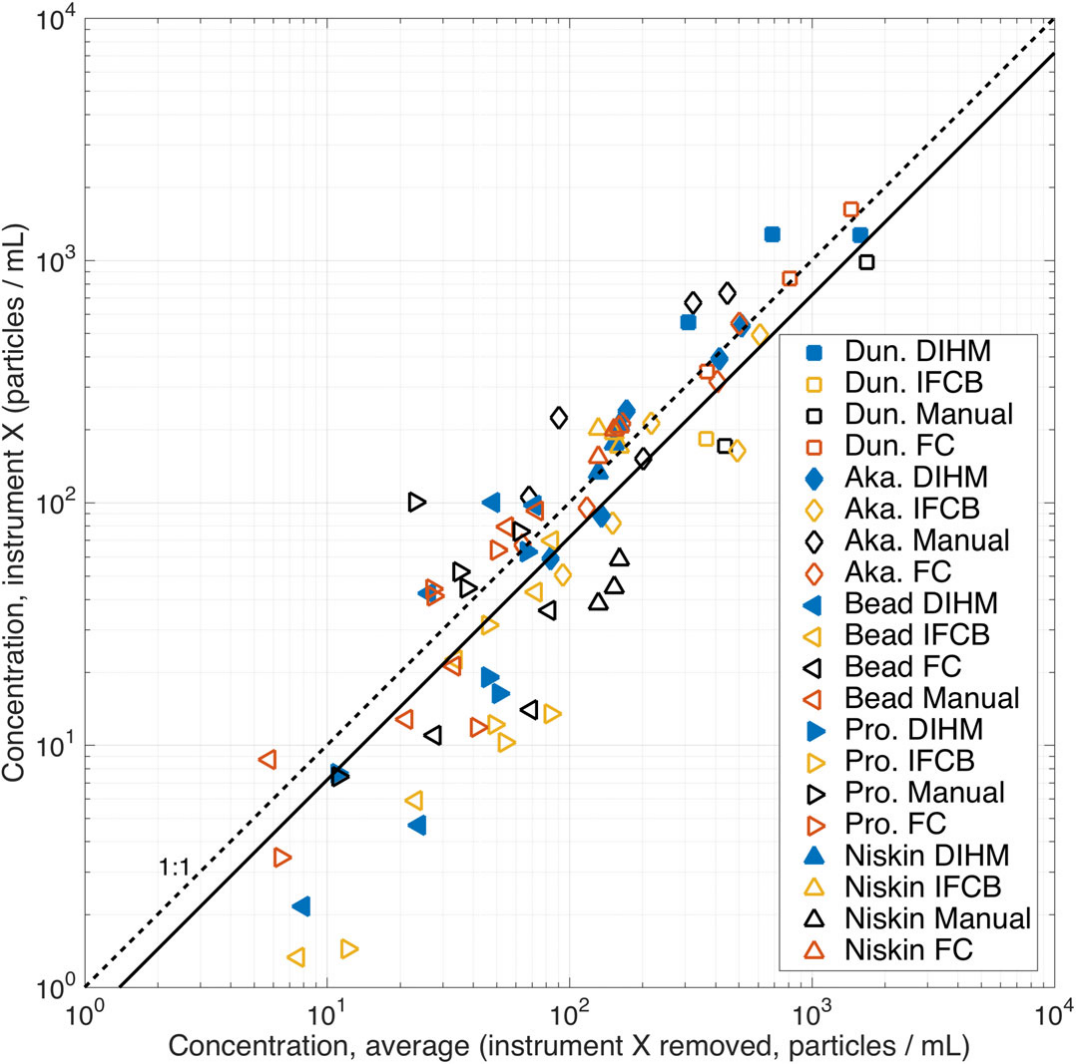
Measured particle concentration intercomparison between each measurement type, for a given particle‐type, plotted against its average (for all other measurements of that sample, excluding the instrument of interest) shows good agreement. “Instrument X” refers to the individual instrument of interest, and these data have been excluded from the individual concentration average calculations to avoid spurious correlations between the data. The sample types include *Dunaliella tertiolecta* (Dun.), *Heterosigma akashiwo* (Aka.), and *Prorocentrum micans* (Pro.), 50 *μ*m microsphere (bead), and environmental samples (Niskin). The 1 : 1 dashed line indicates the slope of the ensemble average of all the concentration measurements. Points above the dashed line over‐estimate abundance relative to the average, while points below the dashed lined under‐estimate abundance relative to the average. Solid line indicates best fit for the equation Conc_inst._ = Conc_avg._ × 10^0.14^, (*r* ^2^ = 0.78).

In the following two sections, we report the quantitative size and concentration intercomparison results for the DIHM, IFCB, FC, and manual microscope counts. Particle size is reported as a size distribution (Fig. 4), where each bin contains the size‐specific concentration. Particle concentration is reported as particles counted divided by volume (Fig. 5). To highlight overall patterns of size and concentration variation for the instrument–instrument combinations, the mean differences in size and concentration for each instrument‐instrument combination are summarized in Table 1.

**Table 1:** The mean difference between each instrument/method for size and concentration. Mean size difference of DIHM − FC is the average of (DIHM − FC) / DIHM for all comparable observations. All comparable observations include the recorded size‐specific concentration for each particle size class. The reported values are the average mean and average standard deviation percent difference for each observed size class, for a total of 229 instrument‐sample size classes. The average mean concentration difference of DIHM − FC is the average of (DIHM − FC) / DIHM for all comparable observations. All comparable observations include the corrected concentration for each particle concentration, for a total of 63 instrument‐sample particle concentrations. A negative mean percentage difference indicates that Instrument A was on average lower than Instrument B. Both laboratory cultures and field data was included in this analysis.

	Mean size difference (*%*)	Mean concentration difference (*%*)
DIHM – FC	−2.7 ± 11	+10.8 ± 57
DIHM – IFCB	−10.5 ± 16	+28.1 ± 42
IFCB – FC	+10.4 ± 31	−67.3 ± 137
DIHM – manual	–	−55.0 ± 148
FC – manual	–	−230 ± 563
IFCB – manual	–	−165.7 ± 245

### Sizing intercomparison

The monoculture PSDs (Fig. 4b–d) show overall good agreement after the DIHM size and concentration corrections steps are applied. For the small, symmetrically shaped *Dunaliella tertiolecta* PSDs (Fig. 4b1–b3), the average size was measured as between 7 and 11 *μ*m for all samples, and the size‐specific concentration range from 100 to 600 cells (mL *μ*m)^−1^. Literature values report the size of *Dunaliella tertiolecta* as between 9 and 11 *μ*m (Tomas 1997). The range of sizes observed varied between 3.5 and 15 *μ*m, the smallest size variation for all monoculture samples. The variation in the size distributions are most apparent at the highest concentration (Fig. 4b3), where the DIHM has a broader distribution compared to the IFCB and FC. The symmetrical *Heterosigma akashiwo* PSD (Fig. 4c1–c5) showed the strongest correlation between the three instruments for all the samples. The range of sizes observed varied between 10 and 25 *μ*m, the second most variation in size for all monoculture samples. Literature values report the size of *Heterosigma akashiwo* as between 15 and 25 *μ*m (Tomas 1997). The asymmetrically shaped *Prorocentrum micans* PSD (Fig. 4d1–d5) showed the weakest correlation between the three instruments for all the samples. This might be described in part by the irregular motion observed of the particles in laminar flow, which tended project a wide range of silhouettes. Literature values report the size of *Prorocentrum micans* as 35 to 70 *μ*m (Tomas 1997).

The Niskin Bottle PSDs (Fig. 4e) show overall good agreement after the DIHM size and concentration corrections steps are applied to the surface, base of the mixed layer, and deep chlorophyll maxima collections. The minimum bin size was 10 *μm* and the maximum bin width was 120 *μm* for logarithmically spaced bin widths. The particles follow a Junge‐type distribution (Bader 1970), with the greatest size‐specific concentration for the 10 *μ*m size class, and the smallest size‐specific concentration for the 120 *μ*m size class. The average slope of these PSDs were −4.0, −4.0, and −3.6 for the surface, base of the mixed layer, and deep chlorophyll maximum, respectively.

### Concentration intercomparison

The overall concentration results (Fig. 5) illustrate the range of concentration values measured by all methods, for all 84 permutations of instrument, sample, and concentration. Potential autocorrelation was removed by removing the instrument data that were included in the average. The minimum observed concentration was 1.3 particles mL^−1^ and the maximum observed concentration was 1624 particles mL^−1^. The average of all measurements for a given sample (excluding the instrument of interest, plotted as the dependent variable along the x‐axis) provides a convenient means to assess which measurements are lower or higher than the average. Measurements that fall along the 1 : 1 concentration average (dashed line) indicate that the overall concentration for that sample or instrument is close to the average of all samples. In general, there appears to be greater variation from the average concentration for low concentrations (∼ 10^1^ cells mL^−1^), and high convergence on the sample average for middle (∼ 10^2^ cells mL^−1^) and high (∼ 10^3^ cells mL^−1^) concentrations. Sample sizes for low concentration samples tended to be low, with fewer than 10 observations for some samples, suggesting low statistical confidence in these results. Sample sizes for middle and high concentrations ranged between 10^3^ and 10^6^ observations.

**Fig 6:**
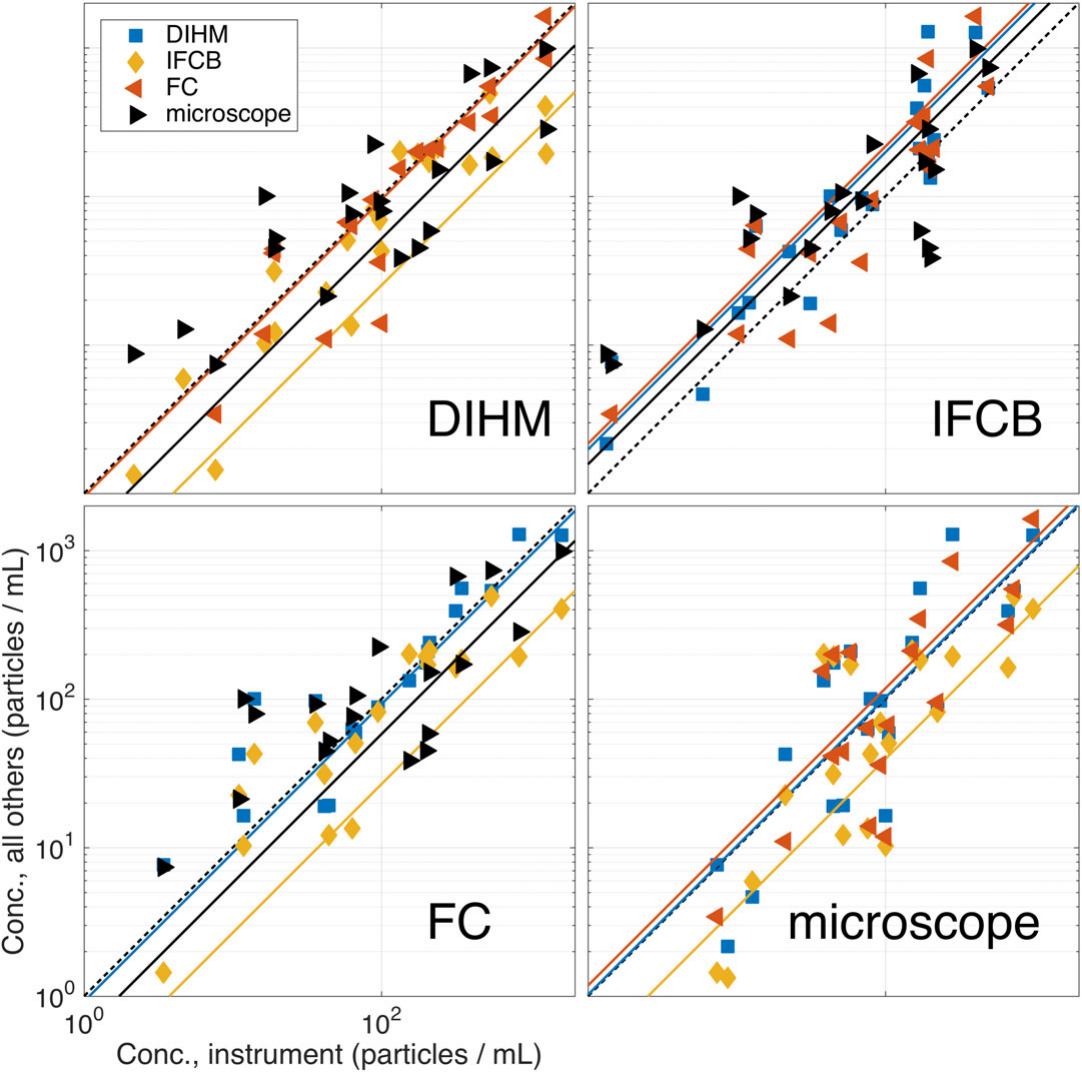
On the x‐axis for each of these panels is the concentration measured by (**a**) DIHM, (**b**) IFCB, (**c**) FC, and (**d**) manual microscope. On the y‐axis is the concentration measured by each of the other instruments. This figure illustrates the scatter and trend of one instrument relative to another instrument. A linear regression, forced through the intercept (in log–log space) for each series was plotted as a dashed line. The form of these linear regression equations are given as Conc_inst.B_ = Conc_inst.A_ × 10^*m*^. The slope *m* and the *r* ^2^ values are given in the Supplementary Information. A 1 : 1 relationship indicates a match between the concentration observed between the instrument and the mean of all instruments.

Finally, to highlight individual relationships for each instrument–instrument intercomparison, we have also provided correlation diagrams (Fig. 6). This log–log representation also demonstrates a first‐order, linear model for which we might consider interchanging observations between instrument observations schemes for achieving a common measurement framework. This model is fit using a least‐squares approach, and forced through the zero intercept so that predictions converge in non‐negative concentration‐space. For example, to predict how an IFCB would measure particle concentration were it in the place of a DIHM, apply the formula *y* = *x* ^*m*^, where *y* is the predicted IFCB concentration, *m* is the empirically determined slope, and *x* is the observed DIHM concentration. Unlike Fig. 5, these diagrams do not compare the observed value to the average of the other observations.

The results of the intercomparison between DIHM, IFCB, FC, and manual microscope counts show overall good agreement after artifact removal steps, size scaling steps, and concentration scaling steps are applied (Fig. 4). Without applying these postprocessing steps, the DIHM data tend to overestimate particle sizes and underestimate the particle concentrations for reasons discussed in the Discussion section. In brief, we have developed the postprocessing size and concentration correction steps that can be applied to the DIHM data independent of the results reported by the IFCB, FC, and manual microscope counts. Statistical measurement of the intercomparability agreement between the IFCB, FC, manual microscope counts and DIHM lend confidence to these methods and results. In the Discussion section, we summarize sources of error and variation within these results.

## *Assessment*

This series of experiments provide new insight into the intercomparability of quantitative particle size and concentration statistics measured by the DIHM and CCV Pipeline. Specifically, we have shown that good agreement between the DIHM, the IFCB and the FC requires artifact removal, size correction, and concentration correction steps are applied to the DIHM data. The size corrections provided here are relevant to holographic systems with point source illumination. The concentration corrections provided here may be relevant to systems with both point source and collimated illumination. These corrections were developed primarily in a laboratory setting with the intent that they will improve the accuracy and precision of field‐recorded data. This step, essential for data interpretation, may introduce bias as laboratory monocultures are not representative of field‐recorded, mixed communities. Field‐recorded data includes particles of mixed shape and size, variable light field (which may affect image contrast), and variable levels of dissolved media in the water (that impacts image quality). For this reason, we have also included an intercomparison of field recorded, environmental samples collected via Niskin bottles. In the following paragraphs, we discuss our experience with multiple sources of error, including illumination quality and holography‐specific imaging challenges. We conclude with recommendations for interpreting these observations in the face of IFCB, FC, and manual microscope‐specific biases.

### Illumination quality and holography‐specific challenges

We have observed that illumination of the imaging volume has an impact on the detection of objects, so the nature of the interaction of the source illumination and particles merits some discussion. As beam intensity attenuates both radially and axially, objects at the periphery of the volume will receive less illumination than those objects closer to the central axis. In addition, partial shading of objects can occur when shadows cast by foreground objects dim background objects. Dimly illuminated objects will be detected less frequently by the ROI detection algorithm, which uses changes in light intensity to detect object edges. This effect was previously described as a “shadow density” problem by Malek et al. (2004). Hence, object concentration changes as a function of light intensity. Samples with a high density of particles will tend to shade background objects with greater frequency than lower concentration samples. Both nonuniform illumination and the sample concentration contribute to bias in quantitative concentration observations.

Davies et al. mitigated the issue of nonuniform, 2D Gaussian illumination distribution for their collimated scheme by expanding the beam to a diameter much greater than the CCD detector size. This created a more uniform light field on the radial cross section of the camera face (Davies et al. 2015). For point‐source illumination schemes, like the DIHM, beam expansion is an inherent feature of the illumination and simply modifying the distance between the laser and the camera may yield more uniformly illuminated holograms. The issue of object shading is present in both point source and collimated schemes (albeit more so in point‐source schemes), but to our knowledge no previous method for mitigating this feature (other than that presented here) has been published.

In addition to the issues of illumination uniformity and shading, geometric magnification of diffraction patterns across the imaging volume also create challenges for achieving a uniform sample in point‐source schemes. For example, as diffraction patterns cast by objects that are closer to the illumination source spread with greater distance on the camera face than those patterns cast by objects close to the camera face, these far‐traveling diffraction patterns can lead to information loss from wave spreading. As diffraction patterns propagate away from the object, some of this information will inevitably propagate outside the camera viewing area. Thus, we suspect that reconstructed objects near the edges of the hologram experience a degraded image quality. As a related issue, partially illuminated objects, or “ghost” particles (those illuminated but outside the camera area), may cast some shadow on the camera and contribute noise to the raw hologram. These issues can be addressed with increased numeric aperture in the fiber‐optic source (which increases the angle of light spread). Local‐adaptive thresholding could also help address issues with noise in low contrast images, however additional calibrations may be needed to characterize how such a potentially variable parameter impacts the local concentration scaling factors. These enhancements are planned for future 4‐Deep DIHM models (Sergey Missan of 4‐Deep, personal communication 11 March 2019).

Overall, we find that there is a trade‐off between optimal illumination and the geometric position of the object in the illumination volume. Although the DIHM is capable of focusing many more focal planes than a traditional microscope, there still exists a “sweet spot” for maximum image quality and size range detectable. We estimate the volume of the sweet spot is 0.030 mL, or 47% of the entire sampling volume. Note that compared to other holographic imaging systems, such as the LISST‐Holo2 with a reported sampling volume of 1.5 mL, this sampling volume is minuscule. For our system, we found the sweet spot to be located along the central axis, about midway between the laser source and detector. The most degraded regions were far from the laser source and the central axis.

These three challenges of holographic systems, nonuniform illumination, shading, and wave spreading, can be problematic for gaining statistical confidence of particle concentration because not all of the diversity of marine particle shapes and sizes fit within the “sweet spot.” Assuming a random detection probability, larger objects are likely to be detected outside optimal detection regions. We have attempted to mitigate this issue by scaling observations of particles according to where it was detected. This can create a possible bias in size‐spectral slope for objects larger than 500 *μ*m, the minimum diameter of the illumination source. However, to account for the presence of these optimal and nonoptimal detection regions, future object detection algorithms should consider a region‐specific weighted approach to ranking and saving contour scores before dimly illuminated objects are discarded.

For all these reasons, imaging volume illumination quality impacts the quantitative measurement of particle concentration. Similarly, instrument‐ and environment‐specific variables may alter the effect that reconstruction and imaging parameters have on object detection. For example, laser light intensity can change with temperature, and the extent of this variation may change for each individual device. This too would impact the quantitative measurement of particle concentration, and highlights the need for an objective approach to determining intensity thresholds used by the reconstruction algorithm. We recommend DIHM users observe their own spatial distribution biases, and correct for the illumination quality accordingly.

### Additional sources of error

Bottle effects and sample treatments added biases or uncertainty to our measurements. Here, we describe some of the possible pitfalls and our efforts to mitigate these. Photosynthetic cultures that were not able to be processed immediately (average time: 3 h, maximum time: 12 h) were stored in light‐opaque bottles to arrest growth. Cells to be processed by manual counts were preserved using Lugol's solution, however this preservation can cause cell shrinking or swelling (Booth 1987; Menden‐Deuer et al. 2001) and impact size estimation. Dead cells and other detritus may have also played a role in biasing measurements. We recommend some additional analysis and quantification of the organism‐specific growth and mortality rates to further close the error gap between the different instruments.

Efforts were taken to avoid spatial sample partitioning via gravitational settling through resuspension of particles by stirring samples in between runs. However, especially for low density samples, “patchiness” in particle distribution was inevitable. This patchiness may account for some of the variance in the observed concentrations. The remaining variability between instrument concentration measurements is ascribed to the overall biases of the methods including, but not limited to, intake pump flow rates, triggering mechanisms, and flow‐cell flow rates. Buoyant or swimming cells likely dispersed nonuniformly in the sampling volume. Efforts were taken to optimize the instrument settings for each individual culture. Flow cell realignment and focusing in‐between dilution runs was avoided. Finally, an ensemble average of all measurements, where each component is weighted by sample size and variability, provides a final metric to which concentration estimates from the DIHM can be assessed.

### Particle orientation and observed size

The extent to which particles are oriented by the sizer during image capture likely impacts the observed particle size (S7). A comparison of observed particle aspect ratios for the IFCB and DIHM highlights this feature (S8). While the IFCB and FC use a laminar sheath fluid for hydrodynamic focusing to fix the imaging focal depth, this tends to orient particle surfaces with the greatest hydrodynamic resistance into the camera, which may lead to lower (i.e., more ellipsoid) observed aspect ratios. The DIHM has no such sheath fluid, which may lead to higher (i.e., more spherical) observed aspect ratios. Consequently, the DIHM is likely to orient particles such that the minor‐axis cross‐section of the particles was at times oriented to the camera. This may have contributed to a greater range of observed particle sizes of the DIHM over the IFCB and FC. Consequently, for the IFCB and FC, lower aspect ratios tend to be observed for elongate objects when compared to the DIHM. For the spherical microspheres, a similarly high distribution in aspect ratio was observed for both the DIHM and IFCB. This may account for the overall lower ESD recorded by the DIHM. For these reasons, we recommend interpreting DIHM size data as a more uniform sample of the particles' 3D shape over the IFCB and FC.

### Conclusion

Over 1 million particles (phytoplankton monocultures, microbeads, and seawater samples) were analyzed using the HoloSea digital inline holographic microscope. This large volume of samples clearly demonstrated the systematic underestimation of particles near the outer edges of the sample volume (Fig. 3) – a bias that must be corrected to obtain accurate concentrations. The measurements were collected in parallel with a FlowCam, Imaging FlowCytobot, and samples for manual microscope identification. Overall, the DIHM showed an underestimate in ESD of about 3% to 10% compared to these other instruments. However, this underestimate may, at least in part, be explained by the randomized orientation of non‐spherical particles observed by the DIHM compared to the streamlined view obtained by the IFCB and FC. Once corrections were applied, the concentration estimates from the DIHM showed linear agreement comparable to the other techniques. Significant effort was required to characterize the DIHM and tune the CCV pipeline so that we felt confident in the intercomparisons. It is possible, and in fact likely, that instrument to instrument variability, and different environmental conditions (particle concentrations, types or other water clarity issues) will require this level of effort for any new setting. Additional development of the reconstruction and ROI segmentation pipeline may help to yield more consistent results, for example, neural networks have recently been developed for aiding in this noise‐sensitive task (Rivenson et al. 2018). Nonetheless, we look forward to new ocean and aquatic observations made possible by this novel technique.

## *Author Contributions Statement*

B.K. and T.S. created the hologram processing pipeline. I.C., M.O., A.N., B.K., Z.L., and N.W. contributed to the initial conceptualization of the study. N.W., B.K., and M.O. recorded the hologram data. A.N. and I.C. ran samples on the FC and IFCB. Z.L. and S.B. performed manual microscope counts. S.M.D. provided cell monoculture. A.M. and I.C. prepared cell cultures for analysis. N.W. performed the analysis with input from M.O., B.K., and S.M.D. N.W. drafted the paper with contributions from M.O., B.K., and S.M.D.

## *Conflict of Interest*

The authors declare that the research was conducted in the absence of any commercial or financial relationships that could be construed as a potential conflict of interest.

## Footnotes

^1^
http://4-deep.com/

^2^
http://s332746484.websitehome.co.uk/holoproc/

^3^
https://www.sequoiasci.com/product/lisst-holo/

^4^
http://4-deep.com/products/stingray-software/

^5^
https://github.com/VROcean

^6^
https://www.bangslabs.com/

^7^
http://www.bemis.com

^8^
https://www.mathworks.com/products/image.html

^9^
https://www.fluidimaging.com/products/particle-analysis-software

^10^
https://github.com/hsosik/ifcb-analysis

^11^
http://4-deep.com/wp-content/uploads/HoloSeaUserGuide.pdf

^12^
https://mclanelabs.com/wp-content/uploads/McLane-IFCB-Datasheet.pdf

^13^
https://info.fluidimaging.com/hs-fs/hub/300163/file-1858782518-pdf/documents/FlowCAM-Aquatic-Applications-Brochure.pdf
